# Histopathological imaging database for oral cancer analysis

**DOI:** 10.1016/j.dib.2020.105114

**Published:** 2020-01-13

**Authors:** Tabassum Yesmin Rahman, Lipi B. Mahanta, Anup K. Das, Jagannath D. Sarma

**Affiliations:** a Department of Computer Science & IT, Cotton University, Panbazar, Guwahati, Assam, 781001, India; b Centre for Computational and Numerical Sciences Division, Institute of Advanced Study in Science and Technology, Guwahati, Assam, 781036, India; c Arya Wellness Centre, GMC Hospital Rd, Near GMDA, Bhangagarh, Guwahati, Assam, 781032, India; d Dr B Borooah Cancer Institute, Guwahati, Assam, 781016, India

**Keywords:** Oral cancer, Histopathology, OSCC, Biopsy slides, 100x, 400x

## Abstract

The repository is composed of 1224 images divided into two sets of images with two different resolutions. First set consists of 89 histopathological images with the normal epithelium of the oral cavity and 439 images of Oral Squamous Cell Carcinoma (OSCC) in 100x magnification. The second set consists of 201 images with the normal epithelium of the oral cavity and 495 histopathological images of OSCC in 400x magnification. The images were captured using a Leica ICC50 HD microscope from Hematoxyline and Eosin (H&E) stained tissue slides collected, prepared and catalogued by medical experts from 230 patients. A subset of 269 images from the second data set was used to detect OSCC based on textural features [1]. Histopathology plays a very important role in diagnosing a disease. It is the investigation of biological tissues to detect the presence of diseased cells in microscopic detail. It usually involves a biopsy. Till date biopsy is the gold-standard test to diagnose cancer. The biopsy slides are examined based on various cytological criteria under a microscope. Therefore, there is a high possibility of not retaining uniformity and ensuring reproducibility in outcomes [2, 3]. Computational diagnostic tools, on the other hand, facilitate objective judgments by making the use of the quantitative measure. This dataset can be utilized in establishing automated diagnostic tool using Artificial Intelligence approaches.

**Table:** Specifications Table

Subject	Computer Science, Computer Vision and Pattern Recognition
Specific subject area	Medical Image Processing, Oral Biopsy Images, Cell segmentation, Cell classification
Type of data	Images
How data were acquired	Images were captured using a Leica DM 750 microscope with camera model ICC50 HD, in 100x (10x objective lens × 10x eyepiece) and 400x (40x objective lens × 10x eyepiece) magnifications (size 2048× 1536 pixels).
Data format	Raw JPG
Parameters for data collection	Images were captured in 100x (10x objective lens × 10x eyepiece) and 400x (40x objective lens × 10x eyepiece) magnifications. The size of the images is 2048 × 1536 pixels.
Description of data collection	Biopsy slides were collected from two reputed healthcare service institutions, Ayursundra Healthcare Pvt. Ltd and Dr B. Borooah Cancer Institute from 230 patients recommended for Oral Biopsy test. The collection period was from October 2016 to November 2017. Images were captured using a Leica DM 750 microscope, model ICC50 HD connected to the camera and a high-configured computer and software. Images were captured in 100× and 400× magnifications.
Data source location	1. Ayursundra Healthcare Pvt. Ltd, Guwahati, Assam, India 2. Dr. B. Borooah Cancer Research Institute (a Regional Cancer Centre recognized by the Government of India), Guwahati, Assam, India
Data accessibility	Rahman, Tabassum Yesmin (2019), “A histopathological image repository of the normal epithelium of Oral Cavity and Oral Squamous Cell Carcinoma”, Mendeley Data, v1. https://doi.org/10.17632/ftmp4cvtmb.1 The link to the image dataset in GitHub: https://github.com/Tabassum2019/A-histopathological-image-repository-of-normal-epithelium-of-Oral-Cavity-and-OSCC/blob/master/README.md
Related research article	Rahman T. Y., Mahanta L. B., Chakraborty C., Das A. K., Sarma J. D., “*Textural pattern classification for oral squamous cell carcinoma.”* Journal of Microscopy, 269 (1), 85–93, (2017) and Rahman T. Y., Mahanta L. B., Das A. K., Sarma J. D., "Automated oral squamous cell carcinoma identification using shape, texture and color features of whole image strips." Tissue and Cell, 63, April 2020, 101322

**Table:** 

**Value of the Data** • This is the first dataset containing histopathological images of the normal epithelium of the oral cavity and OSCC. • These data can be used as a gold standard for histopathological analysis of OSCC. • Researchers can use these data for extracting cytological as well as tissue level features, in image segmentation and also for classification purposes, and aid in establishing an automated diagnostic tool using Artificial Intelligence approaches. • Classification applying deep learning or semantic segmentation tasks can also be implemented by adding/augmenting images in the dataset. • This dataset can be used for a comparative evaluation of one's experimental findings in future when more dataset of such kind is available.

## 1 Data

**Fig. 1:**
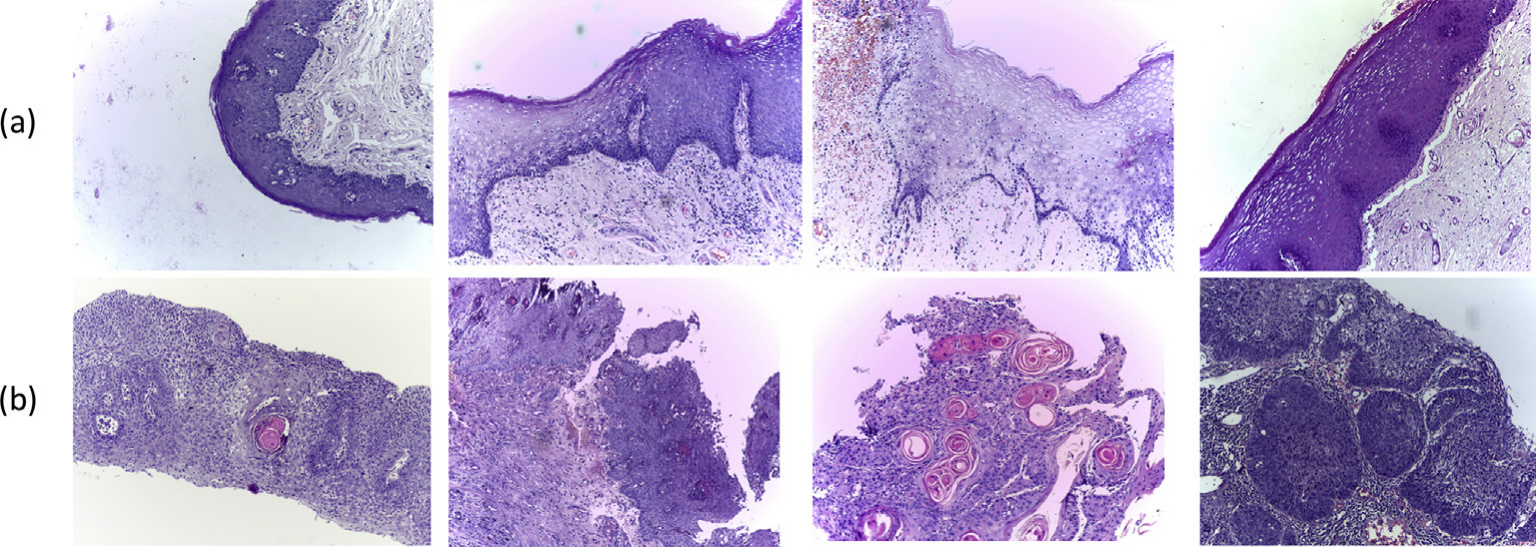
Some images from the first set with (a) normal cells (b) malignant cells.

The data set consists of two sets, each one of which contains images with two categories, normal and abnormal. First set comprises the images captured from the biopsy slides with 100x (10x objective lens × 10x eyepieces) magnification. It consists of total 528 images; out of which of 89 are histopathological images with the normal epithelium of the oral cavity and 439 images are in OSCC category. Fig. 1 depicts some images from the first data set (see Table 1).

**Table 1:** Image details in terms of type, quantity and application scope.

Type	Category	Quantity	Application Scope
100x	Normal OSCC	89 439	1. In architectural level or tissue level analysis
			2. In feature extraction, segmentation and classification purposes
			3. For establishing an automated decision support system
400x	Normal OSCC	201 495	1. In both cell level (for both cell and nucleus) and tissue level analysis
			2. In feature extraction, segmentation and classification
			3. For automated decision support system set up
Total images		1224	

**Fig. 2:**
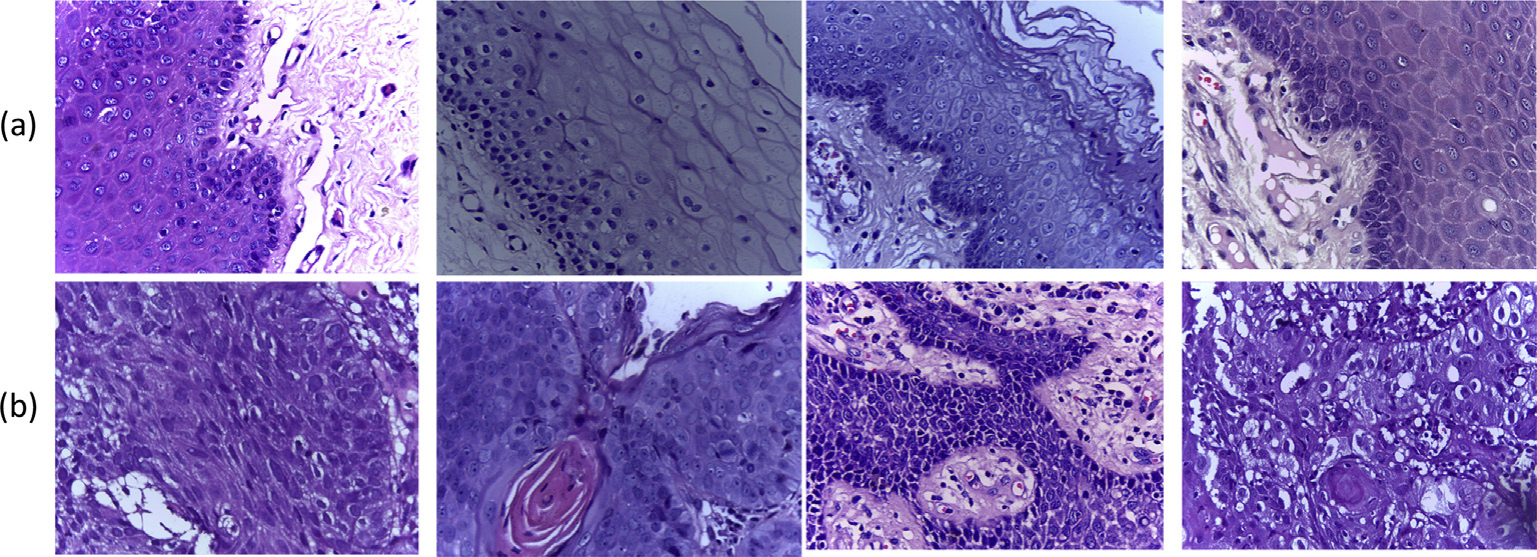
Some images from the second set with (a) normal cells (b) malignant cells.

The images in the second set are of 400x (40x objective lens × 10x eyepieces) magnification. This set contains 696 images, among which 201 images are with normal cell and 495 are with OSCC. Some of the images from this set are shown in Fig. 2. The images from the second data set can be used for both cell level as well as tissue level analysis.

The following table shows the type, category, quantity and application scope of all images:

## 2 Experimental design, materials, and methods

For acquiring the data, i.e. the histopathological images, H&E stained punch biopsy slides were collected from two well known diagnostic centres of the region namely, Ayursundra Healthcare Pvt. Ltd. and Dr B. Borooah Cancer Institute (BBCI) (a Regional Cancer Centre recognized by the Government of India), Guwahati, Assam, India. Patients visiting the organizations with recommendations of oral biopsy tests were included for acquiring the images. The period of collection was from October 2016 to November 2017. The tissue sections belong to the buccal mucosa, as being the dominant area of oral cancer, both globally, nationally and in the specified region. Punch biopsy generally acquires epithelial layer along with some connective tissue layer. Clinician fixed the henceforth-collected biopsies immediately in 4% buffered formalin solution. Following fixation for 48 hours, the fixed tissues were dehydrated in a series of different concentrations of alcohol followed by clearing in xylene and embedding in paraffin wax. Paraffin blocks were then made from the tissues and serial sections were prepared using a microtome at a thickness of 3 μm (micron) on glass slides. The sectioned tissues were then deparaffinised and stained with haematoxylin and eosin using standard protocol. The stained slides were cover slipped with DPX (Dibutylphthalate Polystyrene Xylene) mountant, labelled and examined under a Leica DM 750 microscope (model ICC50 HD).

Images were captured using a camera fitted with the microscope. Captured images are of 100x (10x objective lens × 10x eyepiece) magnification for the first set and 400× (40x objective lens × 10x eyepiece) magnification for a second set of size 2048× 1536 pixels. We have also collected the corresponding pathological reports of the patients, which are used for labelling of the images. These images have a high potential for analysis.

Invasion of the tumour into the basement membrane is a very important architectural feature for diagnosing OSCC. Researchers can use 100x magnified images for architectural or tissue level analysis. These can also be used in feature extraction like shape, texture or colour feature extraction, segmentation of the epithelial layer, invasion of tumour into the basement membrane, or in categorizing images in normal and malignant category considering the whole architecture of the images. 400x magnified images can be used for tissue level analysis, such as in the automated diagnosis of the disease based on the textural feature. A subset of the images with 269 images (134 images with the normal epithelium of the oral cavity and 135 histopathological images of OSCC) was used for an approach to analyze abnormality based on textural features present in OSCC histological slides [1]. Non-uniformity of manual aquisition is a common problem, hence resulting in non-reproducibility of outcomes [2,3]. These have to be dealt with in classification algorithms. Here, applying Histogram and grey-level co-occurrence matrix approaches, textural features of images were extracted and these features were used to categorize the images into the normal and malignant category. 100% classification accuracy was achieved with this approach. These images can also be used for cellular level or nuclear level analysis. One such type of nuclear analysis has beeen caried out by Rahamn et al. [4]. Changes in nucleus such as size, shape etc. play a very important role in differentiating normal cell from a malignant one.

## Transparency document

Transparency document associated with this article can be found in the online version at https://doi.org/10.1111/jmi.12611.

## References

[1] Rahman T.Y., Mahanta L.B., Chakraborty C., Das A.K., Sarma J.D. (2017). Textural pattern classification for oral squamous cell carcinoma. J. Microsc..

[2] Andrion A., Magnani C., Betta P.G., Donna A., Mollo F., Scelsi M. (1995). Malignant mesothelioma of the pleura: interobserver variability. J. Clin. Pathol..

[3] Ismail S.M., Colclough A.B., Dinnen J.S., Eakins D., Evans D.M., Gradwell E. (1989). Observer variation in histopathological diagnosis and grading of cervical intraepithelial neoplasia. Br. Med. J..

[4] Rahman T.Y., Mahanta L.B., Das A.K., Sarma J.D. (April 2020). Automated oral squamous cell carcinoma identification using shape, texture and color features of whole image strips. Tissue Cell.

